# Penetration enhancer-containing spanlastics (PECSs) for transdermal delivery of haloperidol: *in vitro* characterization, *ex vivo* permeation and *in vivo* biodistribution studies

**DOI:** 10.1080/10717544.2017.1410262

**Published:** 2017-12-08

**Authors:** Abdurrahman M. Fahmy, Doaa Ahmed El-Setouhy, Ahmed B. Ibrahim, Basant A. Habib, Saadia A. Tayel, Noha A. Bayoumi

**Affiliations:** ^a^ Department of Pharmaceutics and Industrial Pharmacy, Faculty of Pharmacy, Cairo University Cairo Egypt; ^b^ Labeled Compounds Department, Hot Lab. Center, Egyptian Atomic Energy Authority Cairo Egypt

**Keywords:** Penetration enhancer-containing spanlastics, transdermal *ex vivo* permeation, biodistribution, Haloperidol, technetium-99m

## Abstract

Haloperidol (Hal) is one of the widely used antipsychotic drugs. When orally administered, it suffers from low bioavailability due to hepatic first pass metabolism. This study aimed at developing Hal-loaded penetration enhancer-containing spanlastics (PECSs) to increase transdermal permeation of Hal with sustained release. PECSs were successfully prepared using ethanol injection method showing reasonable values of percentage entrapment efficiency, particle size, polydispersity index and zeta potential. The statistical analysis of the *ex vivo* permeation parameters led to the choice of F1L – made of Span^®^ 60 and Tween^®^ 80 at the weight ratio of 4:1 along with 1% w/v Labrasol^®^ – as the selected formula (SF). SF was formulated into a hydrogel by using 2.5% w/v of HPMC K4M. The hydrogel exhibited good *in vitro* characteristics. Also, it retained its physical and chemical stability for one month in the refrigerator. The radiolabeling of SF showed a maximum yield by mixing of 100 µl of diluted formula with 50 µl saline having 200 MBq of ^99m^Tc and containing 13.6 mg of reducing agent (NaBH_4_) and volume completed to 300 µl by saline at pH 10 for 10 min as reaction time. The biodistribution study showed that the transdermal ^99m^Tc-SF hydrogel exhibited a more sustained release pattern and longer circulation duration with pulsatile behavior in the blood and higher brain levels than the oral ^99m^Tc-SF dispersion. So, transdermal hydrogel of SF may be considered a promising sustained release formula for Hal maintenance therapy with reduced dose size and less frequent administration than oral formula.

## Introduction

Haloperidol (Hal), the dopamine D_2_ receptor antagonist is a widely prescribed typical antipsychotic drug for the acute and maintenance therapy of schizophrenia, mania and other psychiatric disorders (Derendorf, 1995; Dold et al., 2015). Oral tablets and intramuscular injections are the available dosage forms of Hal in the markets. When orally administered, approximately half of the dose is hepatically metabolized leading to low bioavailability (El-Setouhy et al., 2016). Also, the intramuscular route is known to be invasive and inconvenient for many patients.

The transdermal route, then, may be of special interest as a suitable alternative because it's noninvasive and also avoids the hepatic first-pass metabolism. Other advantages of this route include its ability to maintain the drug delivery for long periods of time leading to decreased dosing frequency and higher patient compliance. Also, it can be used for patients who can't swallow tablets and capsules and those who try to crush tablets to facilitate swallowing which is not acceptable in case of controlled release tablets (Tanner & Marks, 2008).

Hal possesses appreciable characteristics which make it a good candidate for transdermal drug delivery. These include a log octanol-to-water partition coefficient of 4.3, molecular weight of 375.9, water solubility of 14 μg/ml (Elgorashi et al., 2008) and low effective therapeutic concentration (2–13 ng/ml) (Volavka et al., 1992). The maximal flux of Hal from aqueous vehicle was theoretically predicted as approximately 0.15 μg/cm^2^ h (Potts & Guy, 1992; Elgorashi et al., 2008). However, Hal is known to be strongly bound to the stratum corneum (SC) leading to limited permeation (Vaddi et al., 2002). Different approaches have been applied to enhance the transdermal permeation of Hal, including the use of proniosomal gels (Azarbayjani et al., 2009), penetration enhancers (PEs) like farnesol (Kang et al., 2005) and limonene (Lim et al., 2006); and finally physical techniques such as iontophoresis (Alvarez-Figueroa et al., 2006).

Penetration enhancer-containing spanlastics (PECSs) combine the use of nanocarriers (spanlastics), which are known to improve the ocular, dermal, and intestinal delivery of drugs (Kakkar & Kaur, 2011; Kakkar & Pal Kaur, 2013; Tayel et al., 2015) along with PEs, with the aim of facilitating the transdermal delivery of Hal.

So, the aim of this work was to formulate PECSs for transdermal delivery of Hal. PECSs were expected to provide enhanced permeation and sustained release for Hal. This would increase the efficacy of Hal with lower dose sizes and less frequent administration than oral route.

## Materials and methods

### Materials

Haloperidol was kindly supplied by Al Kahira Pharmaceutical Company, Cairo, Egypt. Span^®^ 60, methanol of HPLC grade, acetonitrile of HPLC grade and Tetraglycol^®^ were purchased from Sigma Chemical Company USA. Labrasol^®^ and Transcutol^®^ P were a kind gift from Gattefossé, France. Tween^®^ 80, methanol, absolute ethanol, d,l-Lactic acid, propylene glycol, isopropyl alcohol and sodium acetate trihydrate of analytical grade were purchased from El-Nasr Pharmaceutical Chemicals Company, Cairo, Egypt. Hydroxypropyl methylcellulose (HPMC) grade K4M (viscosity approx. 4000 cp) was a kind gift from El-Nile Pharma Company, Cairo, Egypt. Spectra Por^©^ semi-permeable membrane tubing (molecular weight cutoff 12,000–14,000) was purchased from Spectrum Laboratories Inc., CA, USA. Hairless, newborn rat skin was provided by the animal house of Faculty of Pharmacy, Cairo University, Egypt. Technetium-99m was eluted in the form of ^99m^TcO_4_^−^ from ^99^Mo/^99m^Tc generator, Gentech, Turkey.

### Preparation of Hal penetration enhancer-containing spanlastics (PECSs)

Based on previous work of our research team (data under publication), two spanlastic formulae (F1 and F2) were chosen as a foundation for the preparation of PECSs formulae. They contained Span^®^ 60 along with Tween^®^ 80 as an edge activator (EA). F1: 400 mg Span^®^ 60 and 100 mg of Tween^®^ 80 (4:1 weight ratio) and F2: 300 mg of Span^®^ 60 and 200 mg of Tween^®^ 80 (3:2 weight ratio). Each formula had 25 mg of Hal and the total volume was 10 ml. These were prepared using ethanol injection method as described by Kakkar & Kaur (2011). Briefly, Hal and Span^®^ 60 were dissolved in 2 ml ethanol and injected dropwise into a hot (60 °C) Tween^®^ 80 solution. The resulting hydroalcoholic solution was continuously stirred on a magnetic stirrer (Model MSH-20 D; Witeg Labortechnik GmbH Germany) for 30 minutes at 800 rpm to completely evaporate any remaining ethanol and to form Hal-loaded aqueous spanlastic dispersions. Ultrasonic water-bath sonication (Crest ultrasonics Corp., Trenton, NJ, USA) was done for 5 min to obtain a suitable particle size. These formulae were prepared again, but with the incorporation of one of three penetration enhancers (PE): Labrasol^®^, Transcutol^®^ P or Tetraglycol^®^ (Ahad et al., 2009; Mura et al., 2009). These were used at 1% *w/v* concentration (100 mg) (Sinha & Kaur, 2000) in the final dispersion and were dispersed in the edge activator (EA) solution during preparation. Thus, six PECS formulae were obtained.

### *In vitro* characterization of the prepared vesicles

#### Drug content and entrapment efficiency percent (EE%)

Methanol was used to disrupt the spanlastic vesicles and liberate the drug (Maestrelli et al., 2005; Abdelbary, 2011). Total drug content (unentrapped + entrapped) of the prepared formulae was determined by dissolving 0.2 ml of spanlastic dispersion in 25 ml methanol and then measuring the UV absorbance spectrophotometrically at *λ*_max_ of Hal (243 nm) using UV/VIS spectrophotometer (model UV-1601 PC, Shimadzu, Kyoto, Japan). The EE% was determined by filtration of the dispersion using Whatman filter paper (Grade No. 1, 11 µm). Due to its very limited aqueous solubility, the unentrapped Hal was retained over the filter paper (Aburahma & Abdelbary, 2012). Meanwhile, Hal-containing vesicles were obtained in the filtrate. Then, the sample was treated as mentioned for determination of drug content. The EE% was calculated using the following equation:

(1) $$\text{EE}\text{\%}=\frac{\text{amount }\text{of }\text{haloperidol }\text{entrapped }\text{(mg)}}{\text{total }\text{drug }\text{content }\text{(mg)}}$$

#### Determination of particle size (PS), polydispersity index (PDI) and zeta potential (ZP)

The mean PS, PDI and ZP were determined by Zetasizer (Malvern Instrument Ltd., Worcestershire, UK) at 25 °C after suitable dilution with double-distilled water to give a suitable scattering intensity (Scognamiglio et al., 2013). The PDI was used to indicate the degree of particle size homogeneity. The ZP measurement was done in double-distilled water by observing how the vesicles will move in an electrical field (Scognamiglio et al., 2013). Three replicates were taken for each sample, each measured twice.

### *Ex vivo* permeation study of Hal through hairless, newborn rat skin

#### Skin preparation

The protocol of the study was approved by the institutional review board; Research Ethics Committee-Faculty of Pharmacy, Cairo University (REC-FOPCU). Newly born rats were sacrificed and then the dorsal skin was removed. The dermal surface was cleaned using isopropyl alcohol (Kumar et al., 2007). The skin was frozen at −20 °C for a maximum time of 30 days until used for the study (Al-mahallawi et al., 2015).

#### Determination of equilibrium solubility of Hal in 0.03% v/v lactic acid solution

The equilibrium solubility of Hal was determined to know the suitable volume of permeation medium that ensures maintenance of sink conditions. About 10 ml of 0.03% v/v lactic acid solution containing an excess quantity of Hal in tightly capped glass vial were agitated in a shaking water bath (GFL; Gesellschatt Laboratories, Berlin, Germany) set at 50 strokes per minute at 37 °C for 48 h. Then, the solution was filtered through 0.45 μm membrane filter and the filtrate was analyzed spectrophotometrically at the predetermined *λ*_max_ of Hal (248 nm) after suitable dilution (Tenjarla et al., 1998). Experiment was done in triplicate.

#### *Ex vivo* permeation procedure

The permeation of Hal, through excised rat skin, from the two selected spanlastic formulae (F1 and F2), all PECS formulae and the drug solution in propylene glycol (PG) into 0.03% v/v lactic acid solution was assessed (Vaddi et al., 2001). The skin was soaked in phosphate buffer saline for 2 h before the experiment after allowing it to come to room temperature. The skin was mounted on a plastic dialysis tube with the stratum corneum (SC) facing the donor compartment and the dermis fronting the receptor compartment (*n* = 3 per formula). The available permeation area was approximately 1.5 cm^2^. A volume of the formula equivalent to 5 mg Hal was introduced into the donor compartment while the permeation medium was 30 ml of lactic acid solution at 37 ± 0.5 °C stirred at 100 rpm. A preliminary study showed that no drug could be detected for up to 2 h. So, 0.5 mL samples were withdrawn at (2, 4, 8, 12, 24, 36, and 48 h) and immediately replaced by fresh lactic acid solution to maintain constant volume and sink conditions. Samples were analyzed using a validated HPLC method.

#### HPLC determination of Hal

A validated modified HPLC method (Vaddi et al., 2001) was adopted. The HPLC apparatus consisted of: Isocratic pump LC-10 AD and a UV/VIS detector SPD-10 A connected to a C-R6A chromatopac integrator. The analytical column was a reversed phase micro-particulate Shim pack VP-ODS C_18_ column (4.6 × 250 mm) packed with 5 μm particles; all from Shimadzu, Kyoto, Japan. The mobile phase for the calibration curve consisted of acetonitrile: acetate buffer (pH 5) (60:40). The mobile phase was delivered at a flow rate of 1 ml/min. The mobile phase was filtered and sonicated before use. All assays were performed at room temperature and the detection wavelength was 254 nm. Under these conditions, the retention time for Hal was 3.67 min and calibration curve showed good linearity (*R* ^2^ = 0.9998) in the range from 0.1 to 100 µg/ml.

#### Representation and statistical analysis of permeation data

Permeation profiles for different formulae were drawn by plotting the cumulative amount of drug permeated per unit area (*Q*) (µg/cm^2^) against time. The average flux (*J*) (µg/cm^2^ h) was the slope of the linear regression line of the permeation profile (Elgorashi et al., 2008). Statistical analyses for *Q* and *J* at 24, 36 and 48 h were performed. A selected formula (SF) would be chosen based on these statistical analyses results for obtaining the highest permeability.

### Morphology of SF

The morphology of SF was examined by the transmission electron microscope (TEM) (Jeol JEM-1230, Tokyo, Japan) at 80 kV after being negatively stained by 2*% w/v* aqueous phosphotungestic acid solution (Nour et al., 2016).

### Preparation of SF containing hydrogel (SF-gel)

The SF-gel was prepared by sprinkling 250 mg of hydroxypropyl methylcellulose (HPMC) K4M (4000 cp viscosity) slowly in 10 ml of magnetically stirred SF dispersion, to give a final HPMC concentration of 2.5% *w/v*. The dispersion was then refrigerated until no air bubbles or visible clumps were observed.

### *In vitro* characterization of SF-gel

#### Drug content and uniformity

Accurate quantity of the formulation (0.1 ml) was diluted with methanol in a 10 ml volumetric flask. The solution was stirred with a magnetic stirrer with the aid of gentle heat to ensure complete liberation of Hal from the formula. It was then filtered through a 0.45-μm membrane filter and Hal concentration was determined spectrophotometrically after sufficient dilution with methanol. The test was done in triplicate (Saher et al., 2016).

#### pH determination

The pH was measured by dispersing 1 ml of the gel formula in 9 ml of distilled water on a magnetic stirrer. This was followed by immersing the electrode of the pH meter in the diluted formula and allowing it to reach equilibrium for 1 min (Saher et al., 2016; Radwan et al., 2017). The pH was determined in triplicate.

#### *In vitro* release of Hal from SF, SF-gel, and drug solution and kinetic analysis of release data

The release of Hal from SF (1 ml dispersion), SF-gel (1 g) and 0.03% *v/v* lactic acid Hal solution (2.5 ml), all equivalent to 2.5 mg Hal, was studied. The specified amount of each formula, was sealed in presoaked Spectra Por^©^ semi-permeable membrane tubing of 5 cm length and immersed into 50 ml of release medium (0.03% *v/v* lactic acid solution), placed in amber-colored glass bottles, fitted in the horizontal shaker (at 37 °C, 50 strokes per minute, for 24 h). Aliquots of 1 ml each were taken at different time intervals, and replaced immediately with fresh medium. Samples were analyzed spectrophotometrically for drug content (Shamma & Elsayed, 2013). The release trials were performed in triplicate. To determine the appropriate kinetic model, the *in vitro* release data were analyzed according to zero order, first order, second order and third order release models and diffusion-controlled Higuchi mechanism. The coefficient of determination (*R* ^2^) was deduced, where the highest *R* ^2^ referred to the order of release.

#### Rheological studies

The prepared gel was assessed using a rotational cone and plate Brookfield viscometer, spindle CPE-41 at 25 ± 1 °C. About 0.5 g of the tested formula was applied to the plate. The rotational speed ranged from 0.5 to 100 rpm with 10 s interval before proceeding to the next speed. The results were valid only when the torque was within the acceptable range of 10–100%. A Plot of viscosity and shear rate in relation to shear stress is drawn. The power law model was used to study the rheological behavior of SF-gel:

(2) $$\tau \;=\;K\gamma^{n}$$

where *τ* is the shear stress, *γ* is the rate of shear, *K* is the consistency index (s) and *n* is the flow index (dimensionless). For a shear thinning fluid, n is between zero and one while it is one in case of Newtonian system and is larger than one in dilatant system (Gad et al., 2008). Non-Newtonian systems are described using several equations (Bingham’s, Casson’s and Carreau’s equations). Comparison of their *R* ^2^ leads to the type of the non-Newtonian system.

### Effect of storage on drug content and *in vitro* release of SF-gel

To study the effect of storage on SF-gel, it was stored in a tightly capped glass vial and kept refrigerated (4–8 °C) for one month. After 15 days and at the end of the storage period, it was evaluated with respect to its appearance, remaining drug content and Hal release profile. The release profile of the stored SF-gel was compared to the freshly prepared one according to the model independent mathematical approach of Moore & Flanner (1996). The similarity factor (*f_2_*) was calculated according to the following formula:

(3) f2=50 log{[1+(1n)∑t=1n(Rt-Tt)2]-0.5×100}

where *n* is the number of sampling points, *R_t_* and *T_t_* are the mean percent released from reference (fresh) and from test (stored) at time *t*. An *f*_2_ value ≥50 indicate that the release profiles were similar, whereas values less than 50 indicate dissimilarity of release profiles.

### *In vivo* biodistribution study

The experimental protocol of the *in vivo* biodistribution study was approved by the institutional ethical committee, Faculty of Pharmacy, Cairo University. The drug biodistribution was conducted in compliance with the guidelines set by the Egyptian Atomic Energy Authority (EAEA).

#### Animal model

Sixty swiss albino mice (22–27 g each) were kept in polypropylene cages, 5 mice/cage, at 25 ± 1 °C and 45–55% relative humidity with 12 h light/dark cycle and free accessibility to food and water.

#### Preparation of radiolabeled SF and SF-gel

The SF was diluted by addition of 200 µl of SF to 200 µl of 0.1 N HCl and completing volume to 1 ml by distilled water. Diluted SF (dil-SF) was radiolabeled using technetium-99 m (^99m^Tc) by direct-labeling method (El-Setouhy et al., 2016; Nour et al., 2016). Technetium-99 m was eluted as ^99m^TcO_4_^−^ from a ^99^Mo/^99m^Tc generator (Gentech, Turkey). Different factors that affect the radiolabeling process (diluted SF amount, NaBH_4_ amount, pH of reaction medium and reaction time) were studied for choosing conditions to obtain the highest radiolabeling yield and best stability (Motaleb et al., 2011). Experiments studying each factor were done in triplicate.

The radiolabeling process was carried out by adding certain volume (25–200 µl) of dil-SF to 50 µl saline that have 200 MBq of ^99m^Tc and containing certain amount (3–18 mg) of reducing agent NaBH_4_ at pH (3–12) adjusted by 0.1 N HCl, 1 N HCl and 0.1 N NaOH. The volume was completed to 300 µl by saline then spotting on paper chromatography (13 × 1 cm) after certain reaction times (5–120 min) at room temperature. The radiolabeling yield and the *in vitro* stability of the radioactive complex of SF (^99m^Tc-SF) were assessed by paper chromatography (PC) and thin layer chromatography (TLC). Acetone was used as a mobile solvent to evaluate the percent of free ^99m^TcO_4_^−^ while the reduced hydrolyzed ^99m^TcO_2_ (colloid) was determined using ethanol:water:ammonium hydroxide mixture (2:5:1, *v/v/v*). The labeling yield percent of ^99m^Tc-SF was determined as follows: (Sakr et al., 2013a; Ibrahim et al., 2015).

(4) $$\%\text{ labeling }\text{yield}=100-[\%{\text{Free}}^{\text{99m}}{\text{TcO}}_{\text{4}}{}^{-}+\%\text{reduced }{\text{hydrolyzed }}^{\text{99m}}{\text{TcO}}_{2}(\text{colloid})]$$

The transdermal hydrogel of ^99m^Tc-SF (^99m^Tc-SF-gel) was prepared from ^99m^Tc-SF by the same method adopted for the formulation of the SF-gel.

#### *In vivo* biodistribution study of oral ^99m^Tc-SF andtransdermal ^99m^Tc-SF-gel

Mice were assigned randomly to two groups. An appropriate amount of the ^99m^Tc-SF equivalent to 33.3 µg of Hal was administrated orally by gavage to each mouse in group A. The same amount of ^99m^Tc-SF-gel (equivalent to 33.3 µg of Hal) was applied to the dorsal shaved skin of each mouse of group B. A preliminary study showed that no radioactivity could be detected 8 h post oral administration. Thus, at each time point – (0.5–8 h) post oral administration and (0.5–48 h) post transdermal application – a subgroup of three animals from each group was randomly chosen, weighed and sacrificed.

Samples of fresh blood were collected in pre-weighed vials and counted. Blood was assumed to be 7% of the total body weight (Sakr et al., 2013b; Ibrahim et al., 2014). Different tissues/organs including brain, intestines, stomach and liver were separated, washed twice using normal saline solution, and made free from adhering tissue/fluid and weighed. The radioactivity present in each tissue/organ was measured using shielded well-type gamma scintillation counter (scalar ratemeter SR7; Nuclear Enterprises Ltd., Edinburgh, USA). The radiopharmaceutical uptake per gram in each tissue/organ [percentage radioactivity per gram (%R/g)] was calculated as a fraction of applied/administered dose using the following equation (Essa et al., 2015).

(5) $$\%\text{Radioactivity }\text{per }\text{gram }\text{tissue, }\text{organ }\text{or }\text{fluid}=\frac{\left(\text{counts }\text{in }\text{sample}\times 100\right)}{\left(\text{weight }\text{of }\text{sample }(\text{g})\times \text{total }\text{counts }\text{injected}\right)}$$

### Statistical analysis

One-way ANOVA was performed using SPSS^®^ software for comparison of different permeation data, % labeling yield and % radioactivity results. Duncan post hoc test was used where applicable. The level of significance was set at *α* = 0.05.

## Results and discussion

### Preparation of Hal penetration enhancer- containing spanlastics (PECSs)

Hal PECSs were successfully prepared by ethanol injection method. Span^®^ 60, is a lipophilic, nonionic surfactant (HLB =4.7). The saturated, lipophilic alkyl chain in Span^®^ 60 facilitates the formation of mono and/or multi-lamellar vesicles. Tween^®^ 80 and surfactant permeation enhancers (Labrasol^®^ and Transcutol^®^ P) if present promote the elasticity of the vesicles creating systems with disrupted packing characteristics able to squeeze themselves through the pores of the skin (Trotta et al., 2002). Also, being hydrophilic, the penetration enhancers have the ability to temporarily widen the skin’s pore size allowing spanlastics to penetrate deeper (Kakkar & Kaur, 2011).

### *In vitro* characterization of the prepared vesicles

The results of various measurements are listed in Table 1(a). The drug content for all formulae ranged from 97.5 ± 0.3 to 103.9 ± 0.2%. All PECSs were able to incorporate a reasonable amount of Hal. The EE% ranged from 47.5% to 95.4%. In F1L, F1T, F2L and F2T, the EE % decreased from the original corresponding original formulae (F1 and F2), while in F1G and F2G the EE % value did not change. That may be due to the fact that both Labrasol^®^ and Transcutol^®^ P are surfactants (Manconi et al., 2011) and they are largely incorporated in the vesicle bilayer and increase the vesicles permeability thus having adverse effect on EE%, while Tetraglycol^®^ is not essentially a surfactant and its incorporation in the bilayer structure is not to the same degree and most of it remain in the medium (Hao et al., 2002; Ahad et al., 2009). The PS ranged from 189.3 nm to 563.6 nm. This size range is still acceptable for transdermal delivery (Mura et al., 2009; Shamma & Elsayed, 2013; Al-mahallawi et al., 2015). PDI values ranged from 0.39 to 0.76 indicating that the prepared formulae were heterogeneous to a certain degree. ZP values ranged from −30.6 to −38.6 mV indicating good physical stability of the vesicles and that no aggregation is expected to occur (Mura et al., 2009).

**Table 1.** (a) Composition and *in vitro* characterization of F1, F2 and the prepared PECSs and (b) *ex vivo* permeation parameters of F1, F2 and the prepared PECSs compared to Hal solution.

	Composition			*In vitro* measurements^c^			
Formula^a^	Span^®^ 60 (mg)	Tween^®^ 80 (mg)	PE^b^	EE%	PS (nm)	PDI	ZP (mV)
(a)							
F1	400	100	–	95.4 ± 1.7	240.9 ± 10.3	0.39 ± 0.03	−32.1 ± 1.7
F1L	400	100	Labrasol^®^	60.3 ± 3.0	439.5 ± 43.8	0.51 ± 0.12	−37.9 ± 1.0
F1T	400	100	Transcutol^®^	61.1 ± 3.9	563.6 ± 35.1	0.49 ± 0.17	−37.9 ± 2.1
F1G	400	100	Tetraglycol^®^	93.7 ± 2.2	471.3 ± 69.8	0.57 ± 0.18	−38.6 ± 0.8
F2	300	200	–	75.3 ± 9.9	189.3 ± 3.3	0.57 ± 0.10	−30.6 ± 1.5
F2L	300	200	Labrasol^®^	50.5 ± 3.5	195.7 ± 4.0	0.53 ± 0.07	−37.2 ± 1.0
F2T	300	200	Transcutol^®^	47.5 ± 1.1	299.6 ± 8.3	0.76 ± 0.08	−36.8 ± 1.2
F2G	300	200	Tetraglycol^®^	72.5 ± 2.5	363.2 ± 20.7	0.57 ± 0.01	−30.6 ± 2.2
		(*Q*) (μg/cm^2^)^c^			(*J*) (µg/cm^2^/h)^c^		
Formula^a^		*Q*_24_	*Q*_36_	*Q*_48_	*J*_24_	*J*_36_	*J*_48_
(b)							
F1		32.2 ± 9.6	78.2 ± 7.8	184.5 ± 42.7	1.5 ± 0.5	2.2 ± 0.4	3.6 ± 0.8
F1L		93.1 ± 14.8	148.1 ± 3.56	208.8 ± 4.5	4.4 ± 0.7	4.5 ± 0.0	4.6 ± 0.00
F1T		73.9 ± 6.9	120.5 ± 11.8	201.3 ± 13.3	3.5 ± 0.3	3.6 ± 0.3	4.2 ± 0.1
F1G		56.1 ± 13.9	107.3 ± 17.4	204.5 ± 20.8	3.5 ± 1.2	3.6 ± 0.6	4.2 ± 0.0
F2		60.6 ± 13.6	102.9 ± 9.0	158.1 ± 11.6	2.8 ± 0.6	3.1 ± 0.2	3.5 ± 0.2
F2L		86.3 ± 0.3	110.9 ± 23.2	236.4 ± 100.4	3.9 ± 0.1	3.4 ± 0.4	4.7 ± 1.6
F2T		72.6 ± 13.5	93.7 ± 20.8	209.8 ± 90.2	3.4 ± 0.6	3.0 ± 0.6	4.2 ± 1.6
F2G		59.1 ± 0.7	86.1 ± 1.6	147.7 ± 3.0	2.8 ± 0.0	2.7 ± 0.0	3.1 ± 0.1
Hal solution		2.7 ± 2.4	6.1 ± 0.5	16.6 ± 6.3	0.1 ± 0.1	0.2 ± 0.0	0.3 ± 0.0

PECSs: Penetration enhancer-containing spanlastics; PE: penetration enhancer; EE%: percentage entrapment efficiency; PS: particle size; PDI: polydispersity index; ZP: zeta potential.

^a^ All formulae contained 2.5 mg Hal/ ml and total volume was 10 ml.

^b^ PE concentration was 1 % w/v.

^c^ All values are reported as mean ± SD (*n* = 3).

### *Ex vivo* permeation study

#### Determination of equilibrium solubility of Hal in 0.03% v/v lactic acid solution

The equilibrium solubility of Hal in 0.03% v/v lactic acid solution was 1.16 ± 0.08 mg/ml.

#### *Ex vivo* permeation results

**Figure 1.**
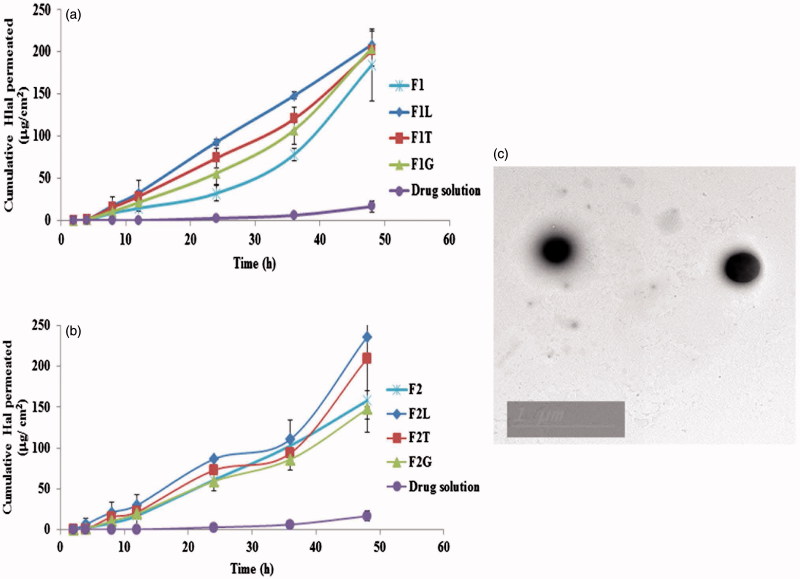
(a) and (b): Permeation plots of Hal from different spanlastics and penetration enhancer containing spanlastics compared to drug solution and (c)Transmission electron microscope image of selected formula.

Permeation plots are shown in Figure 1(a,b). Obviously, Hal was not detected until 4 h and 24 h for different formulae and the drug solution, respectively, suggesting that there may be a lag time. This may be due to the strong binding of Hal to SC (Vaddi et al., 2002) which delays the drug permeation. It's also evident that the drug encapsulation in either conventional spanlastics or PECSs led to earlier detection of the drug in the permeation medium and increased Q at different time intervals in comparison with drug solution. This may be explained in the light of the fact that drug encapsulation in deformable vesicles masks the drug from SC and that the vesicles along with the PEs used enhanced the permeation of drug leading to a net increase in the flux of Hal across the skin. At 24, 36 and 48 h, both (*Q*) and (*J*) were calculated for Hal solution in PG, F1, F2 and PECS formulae. This data is shown in Table 1(b). PECS formulae containing Labrasol^®^ and Transcutol^®^ P showed higher (*Q*) values than the corresponding formulae containing Tetraglycol^®^ at most time intervals. This may be due to the fact that Tetraglycol^®^ is only a secondary PE and its penetration enhancement ability is lower than the two other PEs (Ahad et al., 2009). It was clear that all spanlastic and PECS formulae had significantly higher (*Q*) and (*J*) values than drug solution at all time intervals showing that a significant enhancement of Hal flux across the skin has been achieved. Upon exploring different Duncan’s tests, F1L proved to be the best formula for Hal permeation enhancement. It was of the highest subset in *Q*_24_, *Q*_36_, *Q*_48_, *J*_24_ and *J*_48_. Also, it had significantly higher *J*_36_ than all the other formulae. Thus, considering the *ex vivo* permeation as the most important criterion for selection, F1L was chosen as the SF for further investigation and incorporation into transdermal gel.

### Morphology of SF

The TEM image of SF is shown in Figure 1(c). The vesicles appeared to be perfectly spherical with a particle size comparable to that obtained by Zetasizer.

### Preparation of SF-gel

The hydrogel was successfully prepared, with homogeneous distribution of SF.

### In-vitro *characterization of SF-gel*

#### Drug content and uniformity

The drug content was 98.76 ± 0.23%. Thus, the formula complied with the pharmacopeial limits (Saher et al., 2016).

#### pH determination

The pH of the prepared hydrogel was 5.95 ± 0.03 which is safe to the skin (pH of the skin ranges from 4.5 to 6.5) and wouldn't produce irritation upon application (Schmid-Wendtner & Korting, 2006).

#### *In vitro* release of Hal from SF, SF-gel, and drug solution and kinetic analysis of release data

**Figure 2.**
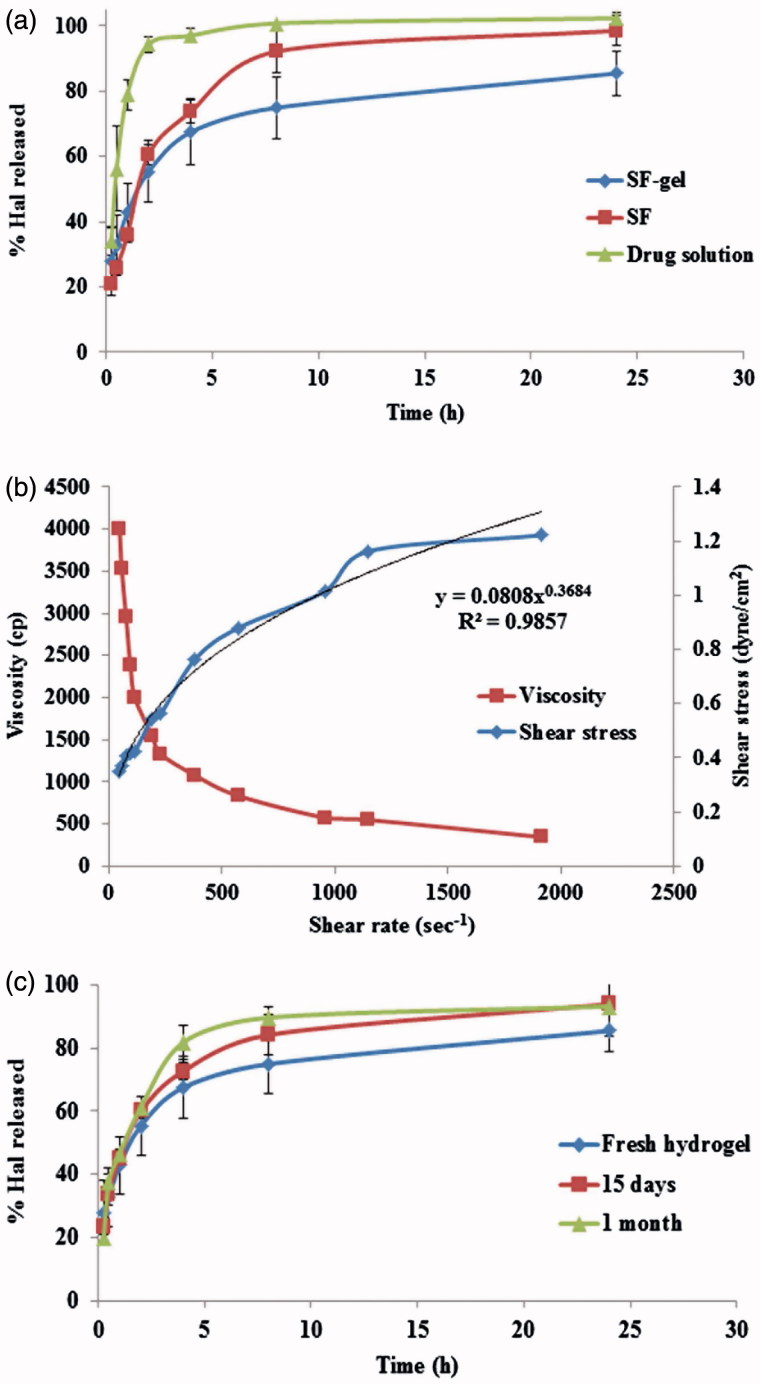
(a) Release profiles of Hal from SF, SF-hydrogel and Hal solution, (b) SF-hydrogel rheological characterization and (c) Effect of storage on Hal release of from SF-hydrogel.

Figure 2(a) shows the release profiles of Hal from SF, SF-gel and drug solution. It's obvious that the incorporation of SF (F1L) into the hydrogel formula has resulted in a more sustained release profile than both SF and, of course the drug solution which released its entire drug content in nearly 2 h. Kinetic analysis showed that the release of Hal from SF-gel fitted best with Higuchi diffusion model showing the highest *R* ^2^.

#### Rheological studies

It was found that SF-gel exhibited shear thinning flow since the viscosity declined upon increasing shear rate (Figure 2(b)). The flow index (*n*) value of the formula was smaller than and far away from one (*n* = 0.3684). Thus the SF-gel follows non-Newtonian and shear thinning behavior according to power law model. Also, it was observed that Carreau’s equation yielded the highest *R* ^2^, indicating pseudoplastic flow. It should be noted that the pseudoplastic flow of hydrogel is highly convenient for topical and transdermal pharmaceutical preparations as at high rate of shear during rubbing the gel on skin, the viscosity of gel decreased so it could spread easily on skin and at low rate of shear it maintained its normal structure (El-Hadidy et al., 2012). The pseudoplastic behavior of the hydrogel could be explained in the light of existence of equilibrium between the effect of random Brownian motion (randomization and entanglement) and the shear induced changes (disentanglement and alignment) which increase by increasing the shear stress. Therefore, upon increasing the shear stress, the polymer molecules are aligned with their long axes in the direction of flow leading to decreased viscosity with increased shear rates. It's also known that Brownian motion gives rise to water entrapment inside the coiled polymer chains. The shear stress releases some of the entrapped water from the dispersed polymer molecules, leading to decrease in their apparent molecular weight and concentration (Ellaithy & El-Shaboury, 2002).

### Effect of storage on drug content and *in-vitro* release of the Hal from the prepared hydrogel

After 15 days and at the end of the 1 month storage period in refrigerator (4–8 °C), SF-gel retained both physical and chemical stability as there was no observed change in its appearance. Also, the estimated drug content of the stored formulation was 97.46 ± 0.36 and 97.17 ± 0.52% after 15 days and 1 month, respectively. There was no significant difference in the drug content of the stored SF-gel when compared to the fresh one at *α* = 0.05. Figure 2(c) shows the similarity in the release profiles of the fresh and stored hydrogels. The difference between the release profiles may be attributed to the possible limited leakage of the drug from PECSs but this was not significant. This was confirmed by the value of similarity factor (*f*_2_ = 64 and 54 after 15 days and 1 month, respectively) which means that these storage conditions had no significant effect on Hal release.

### *In vivo* biodistribution study

#### ^99m^Tc radiolabeling of SF

**Figure 3.**
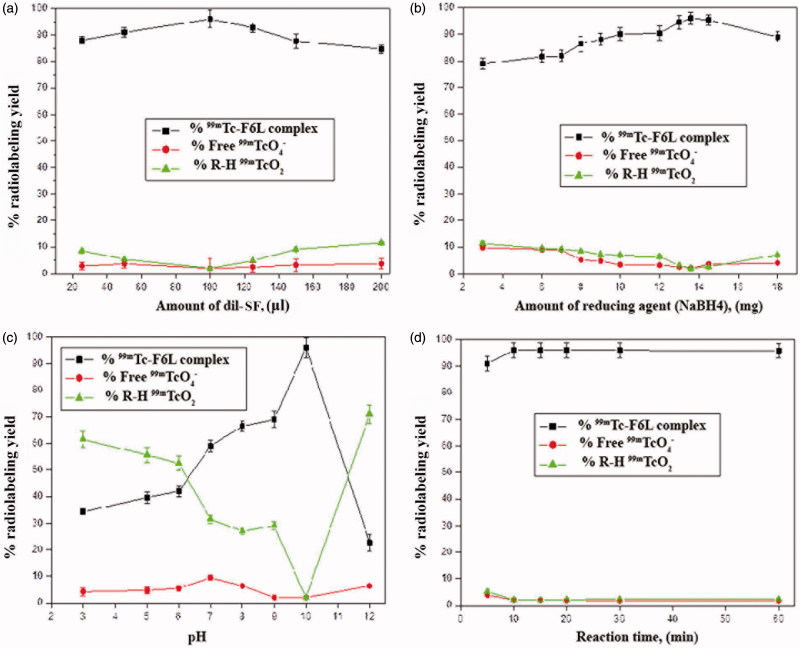
Effect of different variables (a) amount of dil-SF, (b) amount of reducing agent (NaBH4), (c) pH and (d) reaction time – on percent radioactive yield of ^99m^Tc-SF.

The effects of different factors on the radiolabeling yield are shown in Figure 3(a–d). Radiolabeling of SF (F1L) showed maximum radiolabeling yield of (96 ± 0.3%) by mixing 100 µl of dil-SF with 50 µl saline having 200 MBq of ^99m^Tc and containing 13.6 mg of reducing agent (NaBH_4_) and completing volume to 300 µl by saline at pH 10 for 10 min as reaction time. The radioactive complex of SF (^99m^Tc-SF) exhibited good *in vitro* stability up to 48 h.

#### *In vivo* biodistribution study of oral ^99m^Tc-SF andtransdermal ^99m^Tc-SF-gel

**Figure 4.**
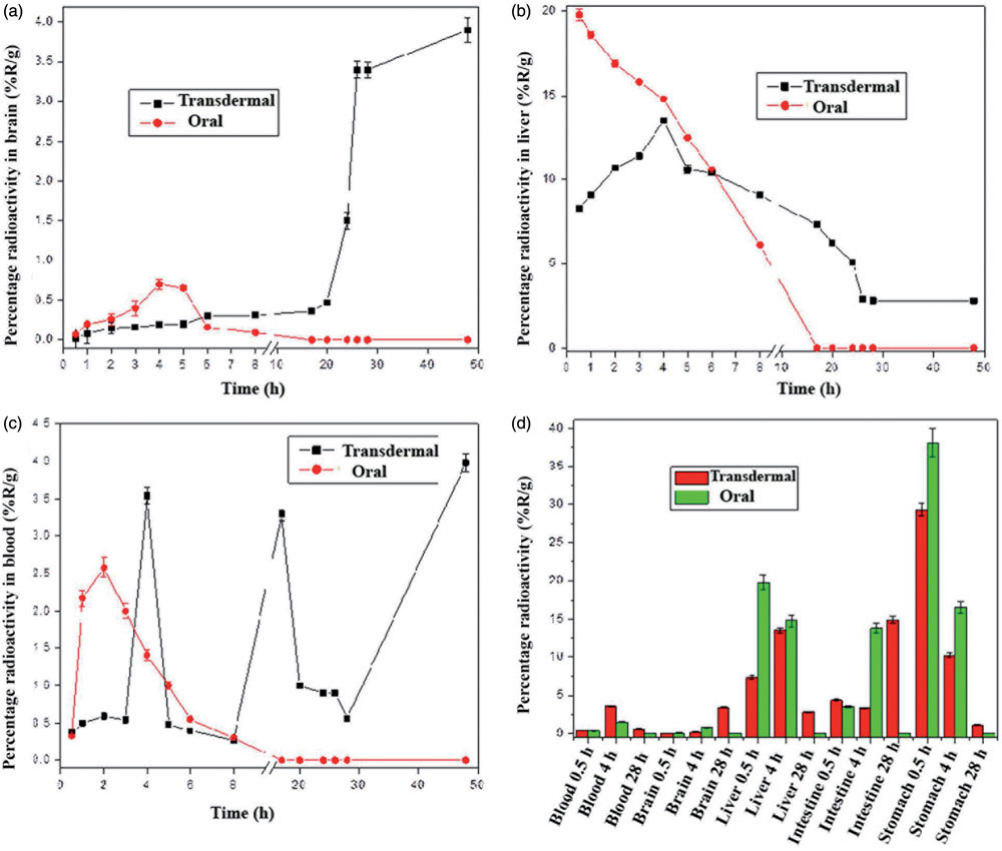
Percentage radioactivity (%R/g) in different body organs and blood at different time points after transdermal application of ^99m^Tc-SF-hydrogel or oral administration of ^99m^Tc-SF.

Figure 4(a–d) shows the different organs uptake and blood levels of radioactive complex after oral ^99m^Tc-SF administration and transdermal ^99m^Tc-SF-gel application at different time intervals. Initially and up to 3 h in the blood and 5 h in the brain, the transdermal application produced lower levels compared to oral administration. Significantly higher brain uptake was achieved 8 h post transdermal hydrogel application than after oral formula administration, (0.31 ± 0.00 vs. 0.09 ± 0.01%R/g), while the blood level for both formulae was not significantly different (0.27 ± 0.01 vs. 0.3 ± 0.06%R/g). This indifference may be explained in the light of the fact that, at 8 h, most of the oral dose is eliminated, leading to lowering of blood level. At the same time, the transdermal dose is beginning to effectively deliver Hal, leading to nearly equal blood level and elevated brain level.

Initially and up to 5 h the liver level of radioactive complex was significantly higher after oral administration. This indicates the extensive first pass metabolism associated with oral dose and explains the undetectability of the radioactive complex 8 h post oral administration in various organs despite of being detected for up to 48 h in case of transdermal application. However, 8 h following transdermal application, the liver level of radioactive complex was significantly higher than after oral administration (9.06 ± 0.05 vs. 6.1 ± 0.10%R/g, respectively). Also the liver, intestines and stomach showed higher radioactive complex levels after transdermal application at 28 h. These detected levels of the radioactive complex after transdermal application in the liver, stomach and the intestine may be because the localization of the complex is both formulation and application-site dependent (Cevc & Blume, 2001). Also, in the intestine there may be a secondary uptake of the transdermally delivered radioactive complex by the intestinal Peyer's patches (Cevc, 1996, 2003).

Comparing the calculated area under the curve from time zero to 8 h (AUC_0–8_) following transdermal and oral routes respectively, it can be observed that; brain, blood and liver levels were significantly lower after transdermal application; with the values of 1.53 vs. 2.39 h%R/g, 6.58 vs. 9.88 h%R/g and 83.89 vs. 106.1 h%R/g, respectively. Such lower systemic and hepatic exposure is desirable for the control of both extrapyramidal side effects (EPS) and hepatic first pass metabolism that follow oral administration (El-Setouhy et al., 2016). Moreover, the transdermal application resulted in a significantly higher *T*_max_ than oral administration (48 h vs. 4 h) to reach maximum brain levels which were significantly much higher than that with oral administration (*C*_max_ = 3.9 ± 0.1 vs. 0.7 ± 0.01%R/g). Deformable vesicles like PECSs are characterized by prolonged circulation times following transdermal application, thus Hal is protected in the blood before its release (Kumar et al., 2012). This is accompanied by accumulation of Hal in the brain (Kornhuber et al., 1999), which can explain these elevated brain levels at late times and favors the use of such formula.

**Figure 5.**
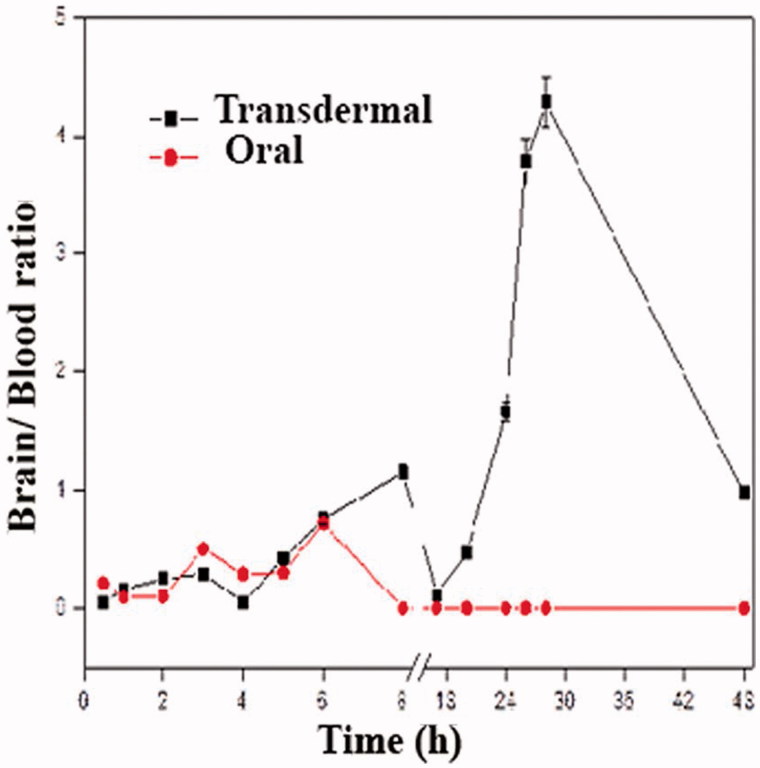
Brain/blood ratio of radioactive complex after transdermal application of ^99m^Tc-SF-hydrogel or oral administration of ^99m^Tc-SF.

For the blood, the transdermal application led to marked fluctuation in blood levels of Hal. It took significantly slower times (*T*_max_) of 4, 17, and 48 h vs. *T*_max-oral_ = 2 h to reach maximum concentrations of 3.54 ± 0.04, 3.3 ± 0.30 and 3.98 ± 0.03 vs. *C*_max-oral_ of 2.58 ± 0.03%R/g. This can be explained in the light of the fact that the skin at the administration surface plays the role of a reservoir for the drug and the SF leading to a pulsatile release pattern which is useful for maintaining high drug levels for prolonged times (Cevc, 2003). The fluctuating blood level post transdermal application also led to different brain/blood ratios at different time intervals with a maximum value of 4.29 post 28 h (Figure 5). This may be attributed to the different rates and relative amounts by which the radioactive complex reaches the systemic and lymphatic circulations post transdermal application (Kota et al., 2007).

Despite the lower brain levels of Hal following transdermal application for 5 h, the drug concentration, based on the percent injected dose delivered 8 h post transdermal application, was calculated to be 99.1 ng/g brain and increases to reach 479.52 ng/g brain and 1246.75 ng/g brain 24 h and 48 h post application, respectively which is higher than therapeutic concentration of Hal (2–13 ng/ml) (Volavka et al., 1992). This may be an evidence of sustained delivery of the drug from the transdermal formula that may also allow for decreasing the size of dose and dosing frequency to minimize the drug’s side effects and increase the patients’ compliance. On the other hand, 8 h post oral administration, the drug concentration was calculated to be 28.77 ng/g brain and afterwards, it was not detectable referring to that the dose has been completely eliminated and the patient would need another dose to maintain a therapeutic drug level. That will decrease the patient’s compliance and will expose him to increased side effects.

## Conclusions

Hal-loaded PECSs were successfully prepared using ethanol injection method. The formulae exhibited reasonable values of EE%, PS, PDI and ZP. The statistical analysis of the *ex vivo* permeation study showed that formula (F1L) had the highest permeation and was chosen as the SF. The TEM image of SF confirmed the spherical morphology of vesicles and that their size was comparable to that obtained by Zetasizer. The HPMC gel of this formula was of suitable viscosity and exhibited pseudoplastic flow, showing Higuchi release mechanism. The biodistribution study of oral ^99m^Tc-SF dispersion and transdermal ^99m^Tc-SF hydrogel showed that the transdermal hydrogel exhibited a more sustained and pulsatile release behavior in the blood with high brain levels. So, transdermal hydrogel of SF may be considered a promising sustained release formula for the maintenance therapy with Hal with reduced dose size and less frequent administration.

## Supplementary Material

IDRD_Doaa_et_al_Supplemental_Content.pdf
