# Supplementary material for: Penetration enhancer-containing spanlastics (PECSs) for transdermal delivery of haloperidol: in vitro characterization, ex vivo permeation and in vivo biodistribution studies
Source: Drug Deliv. 2017 Dec 8;25(1):12–22. doi: 10.1080/10717544.2017.1410262 (PMC6058714; doi:10.1080/10717544.2017.1410262)
Supplement: IDRD_Doaa_et_al_Supplemental_Content.pdf [file IDRD_A_1410262_SM1831.pdf]

In-vitro release kinetic analysis results for selected formula gel (SF-gel)

**Release mechanism**	**Zero order**	**First order**	**Second order**	**Third order**	**Higuchi diffusion**
**Equation**	$C_t = C_0 - K\,t$	$\ln C_t = \ln C_0 - K\,t$	$\frac{x}{a\,(a-x)} = kt$	$\frac{3ax - x^2}{2a^2\,(a-x)^2} = kt$	$Q = \sqrt{D\,t\,(2\,A - Cs)Cs}$
**Terms used**	-(C_t) is the concentration of the drug remaining to be released at time (t)<br>-(C_0) is the initial concentration of the drug<br>-(K) is the release rate constant		-(X) is the concentration of the drug released at time (t)<br>-(a) is the initial concentration of the drug		- (Q) is the amount of drug released per unit area at time (t)<br>- (D) is the diffusion coefficient<br>- (A) is the amount of drug present in the matrix per unit volume<br>- (Cs) is the drug solubility in the matrix.
**Obtained R^2 value**	0.6525	0.5368	0.4280	0.3384	**0.849885**

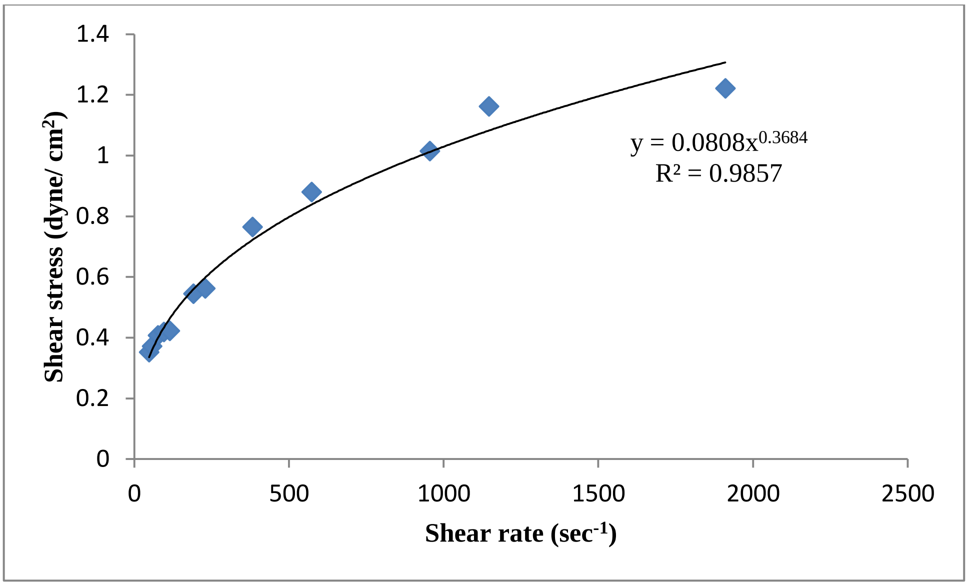

Relation between shear rate and shear stress of SF-gel (Power law plot)

<u>Curves for description of the non –Newtonian system</u>

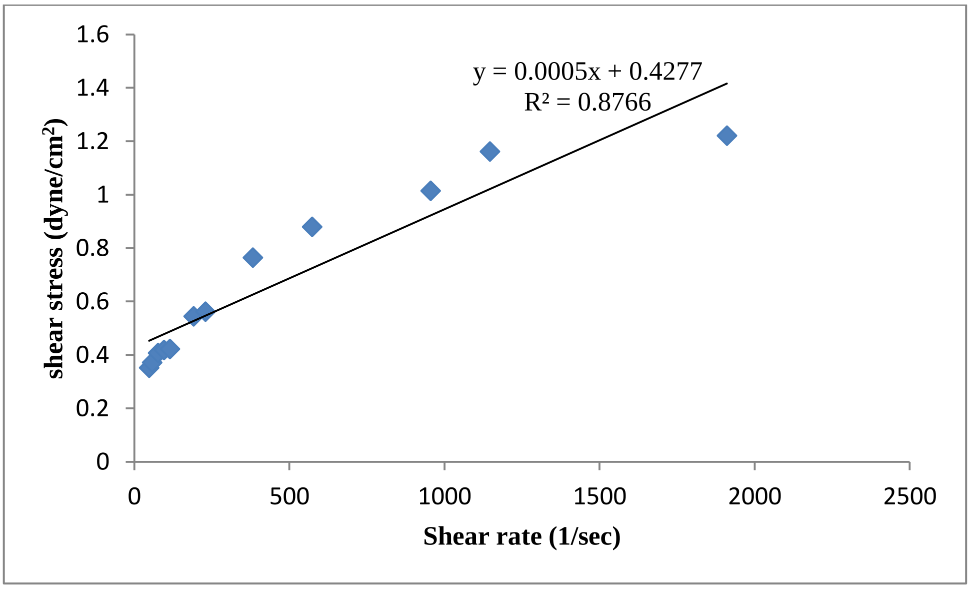

Bingham's plot

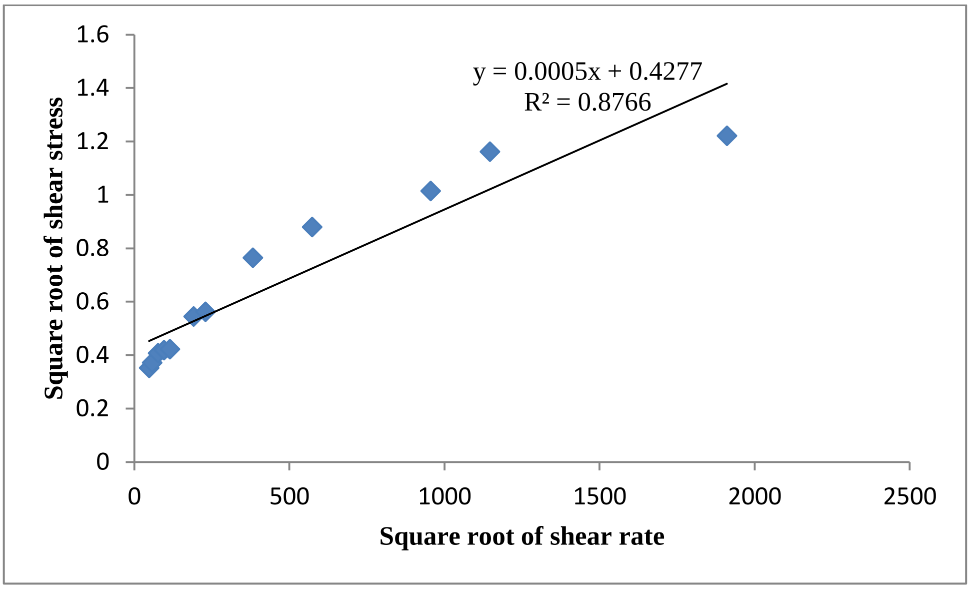

Casson's plot

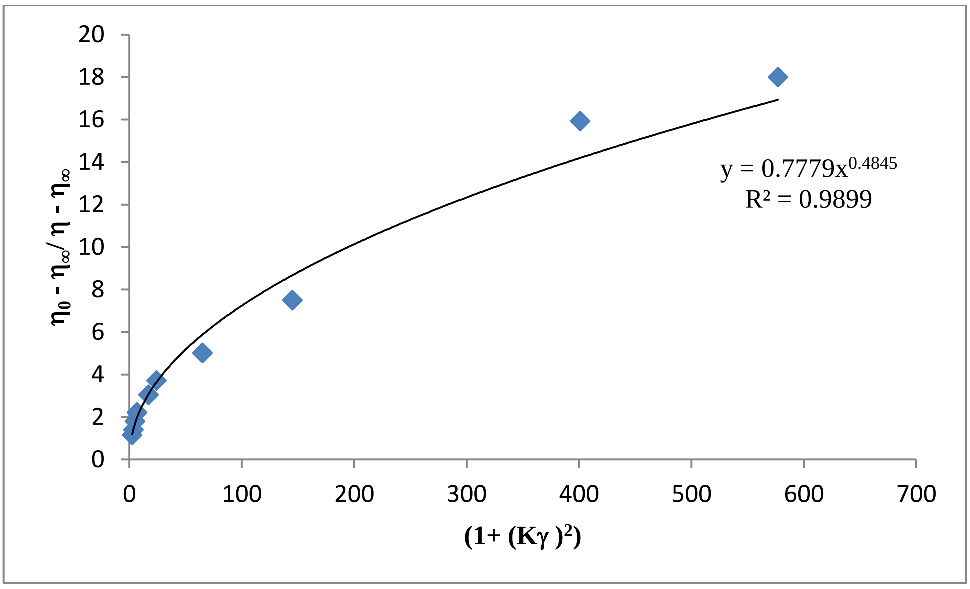

Carreau's plot
